# Effects of implementing a postabortion care strategy in Kinshasa referral hospitals, Democratic Republic of the Congo

**DOI:** 10.1186/s12978-021-01130-x

**Published:** 2021-04-07

**Authors:** Daniel Katuashi Ishoso, Antoinette Tshefu, Thérèse Delvaux, Michèle Dramaix, Guy Mukumpuri, Yves Coppieters

**Affiliations:** 1 Community Health Department, Kinshasa School of Public Health, University of Kinshasa, PO Box11850, Kinshasa1, Democratic Republic of Congo; 2 Public Health Department, Institute of Tropical Medicine, ITM, Antwerp, Belgium; 3 Research Centre of Epidemiology, Biostatistics and Clinical Research, School of Public Health, Université Libre de Bruxelles, Brussels, Belgium; 4 Safe Motherhood Division, National Reproductive Health Program, Ministry of Public Health, Kinshasa, Democratic Republic of Congo; 5 Research Centre “Policies and Health Systems-International Health”, School of Public Health, Université Libre de Bruxelles (ULB), Brussels, Belgium

**Keywords:** Democratic Republic of the Congo, Post abortion care, Intervention, Medical practices

## Abstract

**Objectives:**

To evaluate the effects of the implementation of a postabortion care (PAC) strategy in Kinshasa referral hospitals, this study analyzed the quality of postabortion care services, including postabortion contraception, and the duration of hospitalization.

**Methodology:**

We estimated the effects of the PAC strategy using a quasi-experimental study by evaluating the outcomes of 334 patients with the diagnosis of a complication of induced abortion admitted to 10 hospitals in which the PAC strategy was implemented compared to the same outcomes in 314 patients with the same diagnosis admitted to 10 control facilities from 01/01/2016 to 12/31/2018. In response to government policy, the PAC strategy included the treatment of abortion complications with recommended uterine evacuation technology, the family planning counseling and service provision, linkages with other reproductive health services, including STI evaluation and HIV counseling and/or referral for testing, and partnerships between providers and communities. The information was collected using a questionnaire and stored using open data kit software. We supplemented this information with data abstracted from patient records, facility registries of gynecological obstetrical emergencies, and family planning registries. We analyzed data and developed regression models using STATA15. Thus, we compared changes in use of specific treatments and duration of hospitalization using a "difference-in-differences" analysis.

**Results:**

The implementation of PAC strategy in Kinshasa referral hospitals has resulted in the utilization of WHO recommended uterine evacuation method MVA (29.3% more in the experimental structures, p = 0.025), a significant decline in sharp-curettage (19.3% less, p = 0.132), and a decline in the duration of hospitalization of patients admitted for PAC (1 day less, p = 0.020). We did not observe any change in the use of PAC services, mortality, and the provision of post abortion contraception.

**Conclusion:**

Despite significant improvement in the management of PAC, the uptake in WHO approved technology—namely MVA, and the duration of hospitalization, these outcomes while a significant improvement for DRC, indicate that additional quality improvement strategies for management of PAC and risk-mitigating strategies to reduce barriers to care are required.

## Plain English summary

To evaluate the effects of the implementation of a postabortion care (PAC) strategy in Kinshasa referral hospitals, this study analyzed the quality of postabortion care services, including postabortion contraception, and the duration of hospitalization.

We estimated these effects by evaluating the outcomes of 334 patients with the diagnosis of a complication of induced abortion admitted to 10 hospitals in which the PAC strategy was implemented compared to the same outcomes in 314 patients with the same diagnosis admitted to 10 control facilities from 01/01/2016 to 12/31/2018. The PAC strategy included the treatment of abortion complications with recommended uterine evacuation technology, the family planning counseling and service provision, linkages with other reproductive health services, including STI evaluation and HIV counseling and/or referral for testing, and partnerships between providers and communities. Patient data were collected from patient records, facility registries of gynecological obstetrical emergencies, and family planning registries, and analysed.

The implementation of PAC strategy in Kinshasa referral hospitals has resulted in the utilization of WHO recommended uterine evacuation method MVA (29.3% more in the experimental structures, p = 0.025), a significant decline in sharp-curettage (19.3% less, p = 0.132), and a decline in the duration of hospitalization of patients admitted for PAC (1 day less, p = 0.020). We did not observe any change in the use of PAC services, mortality, and the provision of post abortion contraception.

In conclusion, despite significant improvement in the management of PAC, complications, the uptake in WHO approved technology—namely MVA, and the duration of hospitalization, these outcomes while a significant improvement for DRC, indicate that additional quality improvement strategies for management of PAC and risk-mitigating strategies to reduce barriers to care are required.

## Background

Each year, nearly 73 million abortions are performed worldwide, according to the recent Guttmacher Institute report based on the 2020 Bearak study [1]. Of these abortions, about 25 million are unsafe (performed by persons lacking the necessary skills or in an environment not in conformity with minimal medical standards, or both) and mostly occurred in developing countries, where around 7 million women are hospitalized each year as a result of complications [2, 3]. These complications account for about 7.9% of maternal deaths [4]. Additionally, long-term morbidities occur following unsafe abortions, including subsequent premature births, psychological sequelae, infertility or subfertility, chronic pelvic pain, ectopic pregnancy and spontaneous abortions [5–7].

In the Democratic Republic of Congo (DRC), where legal advances in the practice of abortion are ignored by the population, and by a large majority of healthcare providers who remain committed to article 32 of the code of medical ethics which prohibits this practice unless its indication is the only way to save the life of the mother in danger, the rate of induced abortions is estimated at 55 per 1000 women of childbearing age in Kinshasa its capital [8]. Nearly half of these abortions are followed by complications that include hemorrhage, infection and traumatic injuries (genital trauma and uterine perforation). About 59% of women with a complication of induced abortion seek care in referral hospitals; the remainder seek care in lower level facilities, health centers and dispensaries [8]. Women admitted to referral hospitals for complications of induced abortions account for about 12.8% of all gynecological obstetrical emergencies. Mortality among these women was 5.6%, and half of these deaths occurred after 2 days of hospitalization. These data suggest that some women with complications of induced abortion may be poorly managed [9].

One of the strategic responses to this problem is the implementation of postabortion care (PAC) which consists of emergency treatment for complications related to abortions, family planning counseling and service provision, linkages with other reproductive health services, including STI evaluation and HIV counseling and/or referral for testing, and partnerships between providers and communities [10]. The DRC Ministry of Public Health through the National Program of Reproductive Health, organized a series of training courses in Obstetric and Neonatal Emergency Care (ONEC) with a focus on PAC. Between February and June 2017, a cascade of trainings in PAC targeted healthcare providers in health facilities in Kinshasa. In this manuscript, we report our evaluation of the effects of these courses on the improvement of quality PAC service provision. Specifically, this study analyzed differences in the following parameters before and after the introduction of PAC: (i) the frequency of patients with a complication of induced abortion admitted for postabortion care; (ii) the frequency of the adequate execution of manual vacuum aspiration (MVA and other therapeutic acts; (iii) duration of hospitalization; (iv) deaths related to the complications of induced abortion; and (v) offers of modern postabortion contraceptive methods.

## Methods

### Type of study

This was a quasi-experimental study of the management of postabortion service delivery before and after the implementation of a PAC strategy in intervention hospitals compared to control hospitals. The period before the intervention was from January 1, 2016 to June 30, 2017, and after was from July 1, 2017 to December 31, 2018.

### Setting

This study was conducted in referral hospitals in Kinshasa. Hospitals are designated as referral hospitals by the DRC Ministry of Health based on the provision of certain services. Referral hospitals support and supervise primary care in health centers, train health professionals, and perform operational and implementation research. They typically have more than 100 inpatient beds which equates to about 100 beds for a population of 100,000 inhabitants [11].

The study population only included women presenting to referral hospitals with complications of induced abortion performed elsewhere.

### Package of interventions—implementation of postabortion care (PAC)

The intervention tested in this study was PAC, which includes the treatment of abortion complications with recommended uterine evacuation technology, the family planning counseling and service provision, linkages with other reproductive health services, including STI evaluation and HIV counseling and/or referral for testing, and partnerships between providers and communities. Table 1 presents the activities of the implementation of the PAC intervention, including the main activities, the responsible persons, and the proposed devices.

**Table 1:** Summary of the implementation strategy for PAC

Activities	Main themes	Responsible party	Target	Training materials
Train providers (6 days of training of trainers followed by training of providers in the facilities)	Overview of PAC Learning objectives: How to conduct counseling after an abortion? How to assess the patient for complications? How to treat complications How to ensure preventive measures against infections	NPRH	Providers in health facilities with obstetric and gynecologic services	Trainer and participant PAC manual, demonstration tools (materials and drugs), and video screenings
Community sensitization	Affix PAC wall posters in health facilities Provide PAC advice cards at all health-related activity (prenatal consultations days, vaccination, …)	Staff of health facilities	All visitors of health facilities	Wall posters and PAC boards
Evaluation of patients	Inform the patient about her PAC rights Perform initial assessment to identify emergency conditions Perform a complete clinical examination Request additional examinations	Midwives, doctors	Patients seeking care for complications of abortions	PAC procedures manual, EONC wall charts, consultation form, blood request and delivery note, transfusion monitoring sheet, educational materials (image boxes, advisory boards and others), and patient register
Treatment of patients	Stabilize the patient’s condition Deal urgently with the complications of abortions	Midwives, doctors		
Provide postabortion counseling	Explain to the patient how to take care of her health Psychologically support the patient Provide FP and HIV counseling Advise during follow-up consultations	Midwives, doctors		

*PAC* postabortion care, *NPRH* National Program for Reproductive Health, *EONC* emergency obstetric and newborn care, *FP* family planning, *HIV* human immunodeficiency virus

In December 2016, the National Program for Reproductive Health (NPRH) identified the health facilities eligible for PAC implementation in Kinshasa, including the 29 referral hospitals. In February 2017, an initial 6-day training was organized by the NPRH targeting two providers per eligible hospital. In June 2017, additional training sessions under the supervision of the NPRH were organized in the hospitals that participated in the initial training.

### Logic model of the intervention

Our hypothesis was that if the human, financial, and logistical resources are mobilized for the training of the providers in PAC, the sensitization of the population, and the care of the patients with complication of abortion, then the provider clinical skills will be increased, the population sensitized, and patients admitted for an abortion complication will be properly managed. With these achievements, the outcomes of PAC will be improved, the duration of hospitalization, and maternal deaths due to complications of induced abortion will be reduced.

### Criteria for the selection of intervention and control hospitals

From the original 29 hospitals, ten intervention hospitals were selected because they had completed the entire PAC intervention. This included: (1) declared eligible for PAC implementation by the NPRH in December 2016; (2) represented at the initial training on PAC in February 2017; (3) performed hospital-based training PAC in June 2017 for all providers (midwives and doctors) in the maternity ward. In total, around 100 providers were reached by these trainings in the 10 intervention hospitals.

The intervention hospitals were matched with ten control hospitals. These hospitals were offered the PAC intervention during the study period but did not respond to at least one of the three criterion for the intervention hospitals. Matching was based on type of employer (private, civil state, military state, catholic denominational, protestant denominational, or salvationist denominational) and the type of neighborhood of residence (semi-rural, eccentric, residential, old cities, and planned cities) [12]. Matching according to the type of employer is taken into account because of the difference that appears in the organization and functioning of the health facilities concerned. The state health facilities have an unlimited range of services, including family planning, they are all over-staffed in the city of Kinshasa, with dilapidated equipment, health care providers with little financial motivation and with several other jobs in order to survive. Faith-based health facilities have a range of services with restrictions in terms of modern contraception for some catholic health facilities, they have understaffed staff compared to the clientele, financially motivated and exhausted each time at the end of service, they are relatively well equipped. Private health facilities, on the other hand, have an unlimited range of services like those in the state, have an adequate number of staff in relation to the clientele, are financially motivated, and are relatively well equipped. And the matching by the type of neighborhood of residence aims at the similarity of the population benefiting from the services.

### Data collection

The impact of implementation of the PAC intervention was estimated by examining the care and outcome of all women who were evaluated at study hospitals for complications of induced abortion. Data were abstracted from all medical records and registers for patients with the diagnosis of complications of induced abortion admitted from January 1, 2016 to December 31, 2018. Data were entered into a digital database installed in the smartphones of nurse and physician investigators. The database included sociodemographic, clinical, para-clinical analysis and therapeutic information, and patient outcomes.

### Measurement of the effects of PAC

We measured the effects of the PAC intervention using variables potentially sensitive to the main activities of the implementation of this intervention (Table 2).

**Table 2:** Indicators measuring the effects of PAC

Strategic components of the PAC	Indicators measuring effects	Operational definition
Awareness of the population	Proportion of patients admitted for induced-abortion complications	Proportion of patients diagnosed as having induced-abortion complications among all gynecologic obstetric emergencies
	Proportion of cases of pelviperitonitis	Proportion of cases of pelviperitonitis at admission among all types of induced-abortion complication
	Proportion of other varieties of abortion complications	Proportion of cases of hemorrhage, endometritis, pelvic abscess, and sepsis diagnosed at admission among all induced-abortion complications
Acquisition of clinical skills	Frequency of adequate manual vacuum aspiration	Frequency of intrauterine manual vacuum aspirations performed by a trained provider
	Frequency of dilatation-curettage	Frequency of curettage performed by a trained provider
Treatment of complications of induced abortion	Duration of hospitalization	Number of days between admission and discharge among survivors
	Mortality following complications of induced abortion	Proportion of deaths among patients admitted for an induced-abortion complication
Counseling on family planning	Proportion of patients who received a modern contraceptive method	Proportion of patients who received a modern contraceptive method (contraceptive implant, patch, intrauterine device, contraceptive pill,…) after treatment of the induced-abortion complication

### Data analysis

A comparative description of characteristics was performed at both the individual level (sociodemographic and general clinical characteristics of patients admitted for an induced-abortion related complication) and the structure level to verify the balance between experimental and control structures. At the individual level, a robust standard error linear regression model for cluster sampling was used to compare the mean age of patients in both groups after verification of data normality and homoscedasticity, and logistic regression models with robust standard error for cluster sampling to compare proportions of other categorical variables. At the structure level, the median of percentages by structure accompanied by the minimum and maximum values ​​was used for the "types of provider" and "location of uterine evacuation" variables that were not normally distributed.

For analysis of the effects of the intervention, we generated linear regression models with Robust Standard Errors for cluster sampling (ES and p-values adjusted for clustering), and considered an intra-cluster correlation coefficient being different from 0 for all the variables to be significant. The regression models included the period for the "before” and “after" the intervention for each group. We compared changes in use of specific treatments and duration of hospitalization using a "difference-in-differences" analysis. The models included the group, the period, and the interaction between group and period. For the period of hospitalization which was not normally distributed, quantile regression models were used. An α = 0.05 threshold of significance was chosen.

The data were processed and analyzed using STATA15.

### Ethical considerations

The study was approved by the National Ethics Committee of the Kinshasa School of Public Health (NCE-KSPH). We obtained consent for data collection from administrators at each health facility. The NCE-KSPH waived the need for consent of the participants because data were drawn from the medical records of patients who had either died or had been discharged from the hospital, and they insisted on the anonymity of patients in the collection and analysis of data. We maintained confidentiality by de-identifying all personal health data. The database was password protected, and access was limited to study personnel.

## Results

### Sociodemographic and clinical characteristics of patients

During the study period, 334 women were admitted to intervention hospitals and 314 to control hospitals with the diagnosis of a complication of induced abortion. The sociodemographic characteristics of patients admitted for postabortion care to intervention hospitals compared to control hospitals were not significantly different with exception of parity (Table 3).

**Table 3:** Sociodemographic characteristics of patients

Variables	Intervention hospitals (n = 334)	Control hospitals (n = 314)	p
Average age of the patient (SD)	26.2 (7.4)	25.7 (6.7)	0.643^t^
Adolescent age (≤ 19 years old)			0.891
Yes n (%)	71 (21.5)	60 (20.8)	
No n (%)	260 (78.5)	229 (79.2)	
Total n (%)	331 (100.0)	289 (100.0)	
Marital status			0.281
Single/divorced n (%)	91 (48.9)	117 (57.6)	
Married/cohabiting n (%)	95 (51.1)	86 (42.4)	
Total n (%)	186 (100.0)	203 (100.0)	
Occupational occupation			0.723
Yes n (%)	27 (32.9)	41 (36.0)	
No n (%)	55 (67.1)	73 (64.0)	
Total n (%)	82 (100.0)	114 (100.0)	
Patient parity			0.012
Nulliparous n (%)	91 (41.9)	83 (37.2)	
Primiparous n (%)	43 (19.8)	63 (28.3)	
Multiparous n (%)	83 (38.3)	77 (34.5)	
Total n (%)	217 (100.0)	223 (100.0)	

With SD = standard deviation; n = number of subjects; p = p-value, and ^t^ = t test

At the individual level, the clinical characteristics of patients admitted for postabortion care were not significantly different between the intervention and control hospitals. However, at the structural level, there were differences in the type of providers and location of uterine evacuation (Table 4).

**Table 4:** Clinical characteristics of patients with a complication of induced abortion admitted during the study period for postabortion care

Variable	Intervention hospitals (n = 334)	Control hospitals (n = 314)	p
Individual patient level characteristics^a^			
Bleeding			0.990
Yes n (%)	280 (92.7)	264 (92.6)	
No n (%)	22 (7.3)	21 (7.4)	
Total n (%)	302 (100.0)	285 (100.0)	
Fever			0.364
Yes n (%)	144 (59.0)	203 (75.7)	
No n (%)	100 (41.0)	65 (24.3)	
Total n (%)	244 (100.0)	268 (100.0)	
Abdominal pain			0.630
Yes n (%)	213 (83.9)	239 (89.5)	
No n (%)	41 (16.1)	28 (10.5)	
Total n (%)	254 (100.0)	267 (100.0)	
Traumatic lesions			0.902
Yes n (%)	59 (23.4)	56 (24.5)	
No n (%)	193 (76.6)	173 (75.5)	
Total n (%)	252 (100.0)	229 (100.0)	
Nauseating debris			0.418
Yes n (%)	143 (46.6)	163 (59.5)	
No n (%)	164 (53.4)	111 (40.5)	
Total n (%)	307 (100.0)	274 (100.0)	
Hospital level characteristics			
Type of provider			
^a^Midwife	2.3 (0.0–31.3)	14.7 (0.0–50.0)	
^a^General practitioner	85.5 (61.9–100.0)	68.1 (3.6–100.0)	
^a^Specialist doctor	4.5 (0.0–22.7)	7.2 (0.0–92.9)	
Location of uterine evacuation			
^a^Emergency room	2.5 (0.0)	3.9 (0.0–22.2)	
^a^Labor room	0.0 (0.0–61.9)	0 (0.0–96.3)	
^a^Delivery room	83.1 (0.0–100.0)	89.1 (0.0–100.0)	
^a^Operating room	2.5 (0.0–100.0)	3.7 (0.0–17.9)	

^a^Numbers indicate median percent (minimum value–maximum value) and p = p-value

### Management of postabortion care

#### Number of patients with a complication of induced abortion admitted for postabortion care

The intervention did not have a significant effect on the number of patients with a complication of induced abortion admitted for postabortion care (Table 5).

**Table 5:** Patients with a complication of induced abortion admitted for postabortion care during the period before and after the implementation of the PAC strategy

Variable	Before intervention n (%)	After intervention n (%)	Change % (95% CI95)*		p*
GO emergencies					
Intervention hospitals	1322 (10.1%)	1470 (13.6%)	+ 3.5% (− 3.4 à + 10.4%)		0.284^t^
Control hospitals	1625 (10.9%)	1246 (11.0%)	+ 0.1% (− 3.5 à + 3.7%)		0.950^t^
Difference			+ 3.4% (− 3.6 à + 10.4%)		0.328^t^
Pelviperitonitis					
Intervention hospitals	120 (5.0%)	168 (2.4%)	− 2.6% (− 7.9 à + 2.7%)		0.297^t^
Control hospitals	134 (9.7%)	125 (7.2%)	− 2.5% (− 9.8 à + 4.8%)		0.461^t^
Difference			− 0.1% (− 8.3 à + 8.1%)		0.976^t^
Hemorrhage, sepsis, endometritis, or pelvic abscess					
Intervention hospitals	120 (95.0%)	168 (97.6%)	+ 2.6% (− 2.7 à + 7.9%)		0.297^t^
Control hospitals	134 (90.3%)	125 (92.8%)	+ 2.5% (− 4.8 à + 9.8%)		0.461^t^
Difference			+ 0.1% (− 8.1 à + 8.3%)		0.976^t^

With IC = confidence interval; n = number of subjects; p = p-value; ^t^ = t test; GO = gynecology and obstetrics; * = clustered robust standard errors (cluster = structure)

#### PAC management with manual vacuum aspiration

There was a significant increase in the frequency of manual vacuum aspiration (MVA) in the intervention hospitals but not in the control hospitals. Conversely, there was a significant decline in dilation and curettage (D&C) in the intervention hospitals but not in the control hospitals. The difference in changes between the two groups is significant for the MVA (p = 0.025) (Table 6).

**Table 6:** Treatments received for induced abortion complications

Variable	Before intervention n (%)	After intervention n (%)	Change % (CI95%)*	p*
MVA				
Intervention hospitals	101 (24.8%)	133 (57.1%)	+ 32.4% (+ 7.3 à + 57.5%)	0.017^t^
Control hospitals	104 (15.4%)	103 (18.5%)	+ 3.1% (− 9.2 à + 15.3)	0.585^t^
Difference			+ 29.3% (+ 4.2 à + 54.5%)	0.025^t^
Dilatation and curettage				
Intervention hospitals	113 (66.4%)	150 (38.0%)	− 28.4% (− 52.0 à − 4.7%)	0.024^t^
Control hospitals	140 (64.3%)	114 (55.3%)	− 9.0% (− 24.9 à + 6.9)	0.233^t^
Difference			− 19.4% (− 45.1 à + 6.4)	0.132^t^

With IC = confidence interval; n = number of subjects; p = p-value; ^t^ = t test; MVA = Manual Vacuum Aspiration; * = clustered robust standard errors (cluster = structure)

#### Duration of hospitalization among survivors

The median duration of hospitalization was one longer among survivors in control hospitals during the period after the intervention (4 versus 3). The difference in changes between the two groups is significant (p = 0.020) (Table 7).

**Table 7:** Duration of hospitalization (DH) for treatment of induced abortion complications

Variables	Before intervention		After intervention		Change days (CI95%)	p
	n	Med (P25–P75)	n	Med (P25–P75)		
DH among survivors (days)						
Intervention hospitals	103	3d (2–4)	153	3d (2–5)	0 (− 0.8 à + 0.8)	1.000^k^
Control hospitals	110	3d (2–4)	107	4d (2–4)	+ 1 (− 0.2 à + 1.8)	0.013^k^
Difference					− 1 (− 1.8 à − 0.2)	0.020^k^

With IC = confidence interval; n = number of subjects; p = p-value; and ^k^ = quantile regression

#### Deaths due to complications of induced abortion

The intervention did not have significant effects on deaths related to complications of induced abortion (Table 8).

**Table 8:** Deaths due to induced abortion complications, and offer of post abortion contraception

Variable	Before intervention n (%)	After intervention n (%)	Change % (CI95%)*	p*
Lethality				
Intervention hospitals	126 (3.9%)	183 (1.6%)	− 2.3% (− 6.4 à + 1.8)	0.232^t^
Control hospitals	150 (3.3%)	126 (2.4%)	− 0.9% (− 4.3 à + 2.4)	0.536^t^
Difference			− 1.4% (− 6.2 à + 3.4)	0.553^t^
Offer of post abortion contraception				
Intervention hospitals	105 (34.3%)	137 (33.6%)	− 0.7% (− 18.6 à + 17.1)	0.930^t^
Control hospitals	120 (25.8%)	127 (22.0%)	− 3.8% (− 13.3 à + 5.7%)	0.391^t^
Difference			+ 3.1% (− 15.1 à + 21.3)	0.728^t^

With IC = confidence interval; n = number of subjects; p = p-value; ^t^ = t test; ^*^ = clustered robust standard errors (cluster = structure)

#### Provision of postabortion contraception

The intervention did not have significant effects on the offer of postabortion contraception (Table 8).

## Discussion

The objective of this study was to evaluate the effects of the implementation of a PAC strategy on the management of induced-abortion complications in Kinshasa referral hospitals. This national experience, which includes standard PAC strategies (see Table 1), has the particularity of not including an organizational aspect that has proven successful in several West African countries that have implemented PAC [13]. This includes the creation of areas exclusively dedicated to PAC services, as well as the assignment of trained staff to perform MVAs and provide full-time point-of-treatment counseling and FP services. In addition, aspects of financial subsidy for care and external and internal monitoring were not taken into account in this intervention either.

The quasi-experimental methods used for this purpose, with an ex-post evaluation approach based on the double difference (also known as difference in differences or DID), demonstrated that some PAC treatments improved following the intervention. For example, the frequency of MVA increased and dilation-curettage decreased during the study period in the intervention hospitals but not in the control hospitals. Before the intervention, MVA was performed in less than 25% of patients in all structures combined; this percentage rose to more than 55 after the intervention in the experimental structures. At the same time, the dilatation-curettage procedure decreased from 65%, on average, in both groups of structures to 38% in the experimental structures. This change probably resulted from acquisition of clinical skills by providers. This link between task-transfer training and the execution of therapeutic procedures has been reported for other procedures such as cesarean section and hernia repair [14, 15]. The increase in MVA may be an important evolution in care. Evacuation of the uterine contents can be life-saving in the presence of an incomplete abortion (spontaneous or induced) since non-evacuation can lead to more serious complications such as haemorrhage, sepsis and death [16, 17]. Evacuation can be performed using either MVA or dilatation-curettage. MVA is now recommended because it is a safer, faster, less painful and less inexpensive method compared to dilation and curettage. In addition, it can be performed by nurses or midwives and without general anesthesia [18–20]. By contrast, dilation-curettage is less frequently recommended because it usually requires general anesthesia, an operating theater and the presence of a general physician or specialist [18, 19].

Despite improvements in practice in Kinshasa following implementation of the PAC clinical guidance, optimal practice has not reached the level of some provinces in the east part of the DRC, such as South Kivu and North Kivu, where there was more than 90% MVA and less than 10% dilatation-curettage [21]. Similarly, the practice has not reached the level of some other African countries, such as Kenya, with 65% MVA and 8% dilation-curettage in 2015 [22], Cameroon, with 73% MVA and 23% dilation-curettage in 2014 [23], Egypt, Turkey, Peru, Mexico, Nigeria, Ghana, Senegal, Burkina Faso and Guinea [24–29]. In Ethiopia and Senegal particularly, the level of MVA surpassed the level in DRC in 2004 [30, 31].

However, the change that we observed was greater than that observed in other African countries such as Malawi for example where the percentage of MVA rise from 6.9% in 2013 to 17.4% in 2015 with following implementation of a PAC strategy and a corresponding change in dilation-curettage use from 91 to 78.9% during the same period [32]. Modest changes were attributed to defective equipment and a lack of financial support [33]. Similar studies in Zimbabwe and Uganda reported rates of MVA of 12% and 14% and rates of dilatation-curettage of 75% and 69%, respectively [34, 35].

These observations suggest a need to further improve implementation of best PAC service delivery practices in Kinshasa.

The intervention appeared to have a significant effect on duration of hospitalization following PAC. Although the duration remained unchanged at 3 days in the intervention hospitals, the duration of hospitalization increased from 3 to 4 days in the control hospitals during the same time period. However, it is not clear that a majority of patients benefited from implementation of the PAC strategy. The range of duration of hospitalization among women treated for induced-abortion complications in the intervention hospitals is essentially the same as observed in Kinshasa hospitals in 2014 (1–3 days) [9], and this duration is higher than in many other African countries, which are below 24 h [31, 36]. A possible explanation for prolonged duration of hospitalization in Kinshasa hospitals is that patients are detained for lack of payment of hospitalization costs. It should be noted that in practically the whole city of Kinshasa, hospital costs are borne by patients/families.

Regarding the effects of public awareness activities, the use of PAC services by patients with a complication of induced abortion did not change significantly. However, PAC service use increased numerically in the intervention hospitals, reaching a proportion of 13.6% among all gynecological obstetrical emergency facilities (3.4% more than the control hospitals). This trend in intervention hospitals may be the result of the strategy of raising awareness of abortion complications through the use of wall posters and board cards. The level of this indicator remains lower than that of countries such as Nigeria or Somalia [37–39]. There is also a paradox with Nigeria, which has rates of induced abortions and complications that are lower than those in the DRC (33 versus 55 induced abortions per 1000 women of childbearing age and 23 versus 52 complications per 100 induced abortions) [8, 40, 41] but a higher frequency of complications that present at a hospital. This trend raises suspicion of the existence of abortion complications cases that escape the health system.

Regarding the expected result of emergency treatment of abortion-related complications, the mortality among women with these did not fall significantly during the study period. However, a slight downward trend in mortality was observed in both groups of hospitals. This trend may be related to the effects of other interventions that have been implemented in the context of emergency obstetric and neonatal care. The level of this mortality (1.9%) remains lower than that of many other countries who have implemented PAC [42–45].

The offer of modern postabortion contraception also did not significantly change with the intervention; paradoxically, a decrease was observed during the same period. In reviewing the family planning records, 21.3% (74 out of 348) of the patients did not receive modern postabortion contraception. This is mainly a result of the lack of availability of contraceptive supplies and the religious affiliation of some hospitals. This situation contributes to the current problem of the inefficiency and poor uptake of family planning services throughout the country, where the prevalence of modern contraception has remained low at 8% [46]. The uptake is low despite the strategies to increase utilization, including the purchase of inputs supported by the government, the training of health care providers, mass awareness campaigns and the development of draft legislation to introduce legislation favorable to FP [47]. The level of this indicator in present study was lower than that of the provinces of South Kivu and North Kivu [21].

However, this study had limitation: mortality may be underestimated because the records of these cases were sometimes hidden by persons in charge of the hospitals.

## Conclusion

The implementation of the PAC strategy in Kinshasa referral hospitals improved quality of care for patients admitted for postabortion care. The data indicates the hospital duration was decreased and the uptake of MVA, an approved uterine evacuation technology increased while the utilization of sharp curettage 'an obsolete method' decreased.

However, the use of MVA remains lower than in many other African countries that have implemented this strategy. Given the lack of apparent effect on the use of PAC services by patients (attendance at hospitals offering PAC), the mortality due to complications of induced abortion, and the offer of postabortion contraception, further investigation to determine barriers to quality care and improved outcomes (as for example the negative influences of the absence of areas exclusively devoted to PAC services in hospitals, the absence of financial subsidies and monitoring) is justified. Future strategies to overcome these barriers need to be developed.

## Data Availability

The datasets used and analyzed during the present study are available from the corresponding author upon reasonable request.

## Abbreviations

DRC: Democratic Republic of the Congo
CI: Confidence interval
NCE-KSPH: National Ethics Committee of the Kinshasa School of Public Health
MVA: Manual vacuum aspiration
PAC: Post abortion care
n: Number of subjects
SD: Standard deviation
WHO: World Health Organization
p: p-value
