# Correction to: Effects of implementing a postabortion care strategy in Kinshasa referral hospitals, Democratic Republic of the Congo

**DOI:** 10.1186/s12978-021-01205-9

**Published:** 2021-07-22

**Authors:** Daniel Katuashi Ishoso, Antoinette Tshefu, Thérèse Delvaux, Michèle Dramaix, Guy Mukumpuri, Yves Coppieters

**Affiliations:** 1 Community Health Department, Kinshasa School of Public Health, University of Kinshasa, PO Box11850, Kinshasa1, Democratic Republic of Congo; 2 Public Health Department, Institute of Tropical Medicine, ITM, Antwerp, Belgium; 3 Research Centre of Epidemiology, Biostatistics and Clinical Research, School of Public Health, Université Libre de Bruxelles, Brussels, Belgium; 4 Safe Motherhood Division, National Reproductive Health Program, Ministry of Public Health, Kinshasa, Democratic Republic of Congo; 5 Research Centre “Policies and Health Systems-International Health”, School of Public Health, Université Libre de Bruxelles (ULB), Brussels, Belgium

## Correction to: Reprod Health (2021) 18:76 https://doi.org/10.1186/s12978-021-01130-x

Following publication of the original article [1] two errors have been identified in the abstract and the plain English summary.

The incorrect and correct information is listed below; the changes are shown in **bold**.

## Incorrect

Abstract

The implementation of PAC strategy in Kinshasa referral hospitals has resulted in the utilization of WHO recommended uterine evacuation method MVA (29.3% more in the experimental structures, p = 0.025), a **significant** decline in sharp-curettage (19.3% less, p = 0.132), and a decline in the duration of hospitalization of patients admitted for PAC (1 day less, p = 0.020).

Plain English summary

The implementation of PAC strategy in Kinshasa referral hospitals has resulted in the utilization of WHO recommended uterine evacuation method MVA (29.3% more in the experimental structures, p = 0.025), a **significant** decline in sharp-curettage (19.3% less, p = 0.132), and a decline in the duration of hospitalization of patients admitted for PAC (1 day less, p = 0.020).

## Correct

Abstract

The implementation of PAC strategy in Kinshasa referral hospitals has resulted in the utilization of WHO recommended uterine evacuation method MVA (29.3% more in the experimental structures, p = 0.025), a **non-significant** decline in sharp-curettage (19.3% less, p = 0.132), and a decline in the duration of hospitalization of patients admitted for PAC (1 day less, p = 0.020).

Plain English summary

The implementation of PAC strategy in Kinshasa referral hospitals has resulted in the utilization of WHO recommended uterine evacuation method MVA (29.3% more in the experimental structures, p = 0.025), a **non-significant** decline in sharp-curettage (19.3% less, p = 0.132), and a decline in the duration of hospitalization of patients admitted for PAC (1 day less, p = 0.020).
